# Adoptive Immunotherapy in Postoperative Hepatocellular Carcinoma: A Systemic Review

**DOI:** 10.1371/journal.pone.0042879

**Published:** 2012-08-15

**Authors:** Feng Xie, Xinji Zhang, Hui Li, Tao Zheng, Feng Xu, Rongxi Shen, Long Yan, Jiamei Yang, Jia He

**Affiliations:** 1 Department of Health Statistics, The Second Military Medical University, Shanghai, China; 2 Department of Special Treatment and Liver Transplantation, Eastern Hepatobiliary Surgery Hospital, The Second Military Medical University, Shanghai, China; Virginia Commonwealth University, United States of America

## Abstract

**Purpose:**

The effectiveness of immunotherapy for postoperative hepatocellular carcinoma patients is still controversial. To address this issue, we did a systemic review of the literatures and analyzed the data with emphasis on the recurrence and survival.

**Methods:**

We searched six randomized controlled trials that included adoptive immunotherapy in the postoperative management of hepatocellular carcinoma and compared with non-immunotherapy postoperation. A meta-analysis was carried out to examine one- and 3-year recurrence and survival.

**Results:**

The overall analysis revealed significantly reduced risk of 1-year recurrence in patients receiving adoptive immunotherapy (OR = 0.35; 95% CI, 0.17 to 0.71; p = 0.003), in that the risk of 3-year recurrence with a pooled OR estimated at 0.31 (95% CI 0.16 to 0.61; p = 0.001). However, no statistically significant difference was observed for 3-year survival between groups with adoptive immunotherapy and without adjuvant treatment (OR = 0.91; 95% CI, 0.45 to 1.84; P = 0.792).

**Conclusions:**

Adjuvant immunotherapy with cytokine induced killer cells or lymphokine activated killer cells may reduce recurrence in postoperative hepatocellular carcinoma patients, but may not improve survival.

## Introduction

Each stage of cancer development is regulated uniquely by the immune system; whereas full activation of adaptive immune cells at the tumor stage may result in eradication of malignant cells, chronic activation of innate immune cells at sites of premalignant growth may actually enhance tumor development [1]. Higher incidences of hepatocellular carcinoma (HCC) have been reported in chronic liver disease related to viral hepatitis B and C. And HCC patients often have functional deficiency in host adaptive and innate immune responses against the cancer [2]. Immunotherapy is a promising treatment option for HCC by stimulating the immune system to recognize and kill the tumor cells [3].

Immunotherapy mainly includes lymphokine-activated killer (LAK) cells and cytokine-induced killer (CIK) cells, and has evolved from experimental procedures into early clinical studies with encouraging preliminary efficacy towards susceptible autologous and allogeneic tumor cells in both therapeutic and adjuvant settings. First described in the early 1980s, LAK cells are cytotoxic effector lymphocytes whose cytolytic activities are not restricted by major histocompatibility complex (MHC) and have the ability to kill tumor cells and NK-resistant tumor cell lines [4]. CIK cells are generated by polyclonal T effector cells when cultured under cytokine stimulation. CIK cells exhibit potent, non-MHC-restricted cytolytic activities against susceptible tumor cells of both autologous and allogeneic origins [5].

However, the value of immunotherapy for postoperative HCC patients remains controversial, especially in preventing recurrence and prolonging survival [6]. Takayama et al. reported that immunotherapy can lower recurrence and improve recurrence-free outcomes after surgery for HCC [7]. But Kawata et al. reported no statistically significant difference in the survival rate or in the cumulative disease free rate [8]. The current study is a meta-analysis of published randomized controlled trials to investigate the efficacy of adoptive immunotherapy in postoperative hepatocellular carcinoma.

## Methods

### Search strategy and selection criteria

To be included in the meta-analysis, studies must be randomized controlled trials that compared adoptive immunotherapy with no adjuvant treatment in HCC patients who had undergone curative resection.

Relevant studies were identified by searching PubMed (1976 onward), Embase (1966 onward), the Cochrane Center Register of Controlled Trials (no date restriction), Biological Abstracts (no date restriction), Science Citation Index (no date restriction), China National Knowledge Infrastructure (no date restriction), and the Chinese BioMedical Literature Database (no date restriction). Keywords used included “liver neoplasms”, “liver cancer”, “hepatocellular carcinoma”, “resectable”, “operation”, “operative”, “resection”, “hepatectomy”, “postoperative”, “postoperation”, “immunotherapy”, “cytokine induced killer cells”, “tumor infiltrating lymphocytes”, “lymphokine activated killer cells” and “interleukin-2”. We also manually searched the American Society of Clinical Oncology (ASCO) Annual Scientific Meeting proceedings from 2004 to 2011. In addition, reference lists of the trials selected before and relevant reviews were examined for other eligible trials. We also searched http://www.ClinicalTrials.gov website for the information of prospective and ongoing trials. No language restriction was applied.

### Data extraction and quality assessment

Data extraction was independently conducted by two reviewers (Feng Xie and Xinji Zhang) using a standardized approach. Disagreement was adjudicated by a third reviewer (Hui Li) after referring back to the original publications. The following information was obtained from each source article: year of publication, number of patients, sex, cirrhosis rate, alpha-fetoprotein levels, Child–Pugh class, operative method, immunotherapy regimen, number of patients assessable for 1- and 3- year recurrence, and number of patients assessable for 3-year overall survival.

The modified 10-point Jadad scale [9], [10] was used to assess the quality of the trials based on the reporting of the study methods and results, namely allocation sequence generation, allocation concealment, double blinding, description of protocol deviations, withdrawals, and dropouts, and efficacy of randomization.

### Statistical analysis

The analysis was carried out by pair-wise comparison of the immunotherapy-containing arms of the identified trials with the respective non-immunotherapy arms. One study [15] included in the meta-analysis had a four-arm design: it compared two regimens at two different approaches; therefore, each immunotherapy arm was paired to its natural counterpart defined by the matching treatment approaches.

Treatment effects are reflected by odds ratios (ORs) for recurrence and 1-year survival. To calculate the pooled odds ratio (OR), the number of recurrence or survival in each arm were extracted from each study and combined using a method reported by Mantel and Haenszel [11]. A pooled OR<1 indicated lower recurrence or lower survival in the immunotherapy arm. To evaluate whether the results of the studies were homogeneous, we used the Cochran's Q test (considered significant for P<0.10) [12]. It is a chi-squares test with df equal to the number of studies minus one, and tests the null hypothesis that the difference between the study estimates of OR is due to chance. We also calculated the quantity I^2^ [13], [14] that describes the percentage of variation across studies that is due to heterogeneity rather than chance. I^2^ values of 25%, 50%, and 75% were used as evidence of low, moderate, and high heterogeneity, respectively. The OR was calculated with a fixed-effect model when no statistically significant heterogeneity existed; otherwise, a random-effect model was employed. All the reported p values were two-sided. P values at <0.05 were regarded as statistically significant. Statistic analyses were carried out using STATA 11.0.

## Results

**Figure 1:**
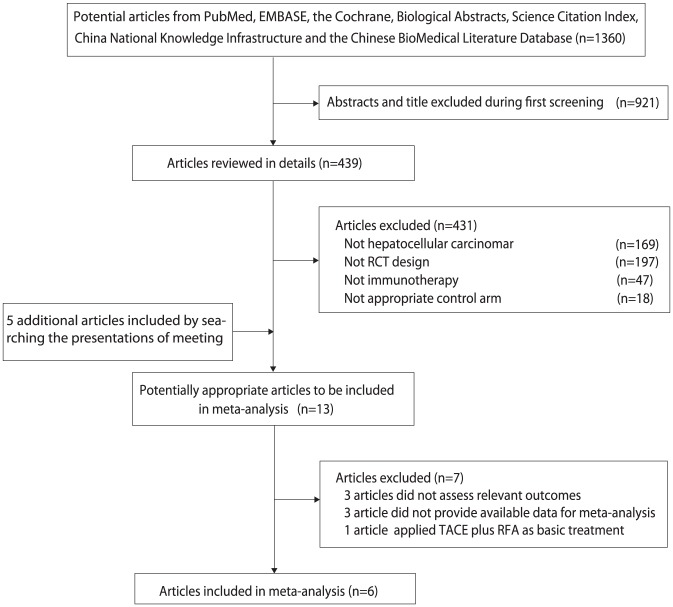
Identification process for eligible studies.

We identified 6 randomized controlled trials [7]–[8], [15]–[18] including 494 patients using the strategy summarized in Figure 1. Of these 6 trials, 4 trials were conducted in mainland China; the remaining two were conducted in Japan. All used resection as basic treatment before adoptive immunotherapy. Two trials used CIK as adoptive immunotherapy, one used CIK plus interleukin-2 (IL-2), and the remaining three used LAK plus IL-2. The characteristics of each study are listed in Table 1.

**Table 1:** Characteristics of included trials.

Authors and Year	Number of Patients	operative method	Immunotherapy method	Male(%)	Cirrhosis(%)	Child-Pugh classification A(%)	AFP> = 400(%)
Dong et al. (2009)	127	Radical resection	CIK+IL-2(via vein;1.0×10^10^ to 2.0×10^10^; 3 infusions)	31(75.6)	34(82.9)	34(82.9)	NR
			CIK+IL-2(via vein;1.0×10^10^ to 2.0×10^10^;6 infusions)	33(74.4)	34(79.1)	34(79.1)	NR
			support therapy	34(79.1)	34(79.1)	34(79.1)	NR
Luet et al. (2008)	30	Non-radical resection	CIK(via vein;1.6×10^10^ cells; 4 infusions)	NR	NR	NR	NR
			support therapy	NR	NR	NR	NR
Zhou et al. (2002)	121	Non-radical resection	immunotherapy +chemotherapy: LAK+IL-2+ chemotherapy ( via artery or both artery and vein; most 4 infusions,some 8 infusions)	NR	NR	NR	NR
			chemotherapy	NR	NR	NR	NR
			Immunotherapy: LAK+IL-2( via artery or both artery and vein; most 4 infusions,some 8 infusions)	NR	NR	NR	NR
			support therapy	NR	NR	NR	NR
Takayama et al.(2000)	150	Radical resection	CIK (via vein,>3×10^10^ cells ; 5 infusions)	NR	35(46.1)	54(71.1)	18(23.9)
			support therapy	NR	38(51.4)	50(67.6)	17(23.0)
Xie et al. (2000)	42	Radical resection	LAK+IL-2+TACE(via artery;1.0×10^9^ cells; 1 infusion)	16(76.2)	NR	NR	NR
			transhepatic arterial chemoembolization	15(71.4)	NR	NR	NR
Kawata et al. (1995)	24	Non-radical resection	LAK+IL-2+adriamycin(via artery; 9.45±4.6×10^9^ cells; 2–3 infusions )	10(83.3)	9(75.0)	NR	NR
			adriamycin	11(91.7)	5(41.7)	NR	NR

Abbreviations: NR, not reported.

The Jadad score was 6 for one trial, 5 for two trials , 3 in two trials, and 2 in the remaining one trial.

### 1-year recurrence

**Figure 2:**
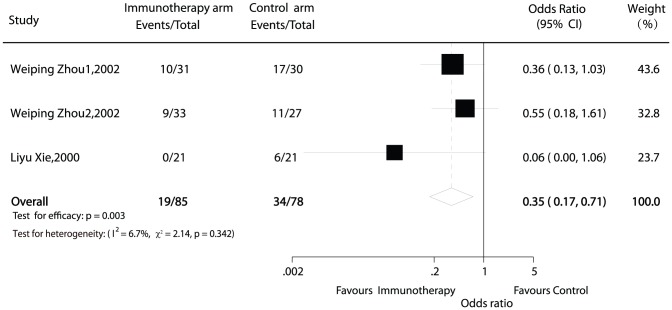
Comparison of 1-year recurrence between hepatocellular carcinomatherapy and non-immunotherapy postoperation therapy.

Information on 1-year recurrence was available in 2 trials [15], [16] contained 163 patients (85 patients received immunotherapy). In each protocol, HCC recurred in 19 (22.3%) adoptive immunotherapy patients compared with 34 (43.6%) controls within 1 year. Only one of the trials showed lower recurrence for adoptive immunotherapy patients while the others did not found difference between the two groups. The estimated pooled OR for both 2 trials shows a highly significant reduction of the risk of recurrence for patients receiving adoptive immunotherapy (OR = 0.35; 95% CI, 0.17 to 0.71; p = 0.003; Fig. 2). The Cochran's Q test had a p value of 0.342 and the corresponding quantity I^2^ was 6.7%, indicating that the degree of variability between trials was consistent with what would be expected to occur by chance alone.

### 3-year recurrence

**Figure 3:**
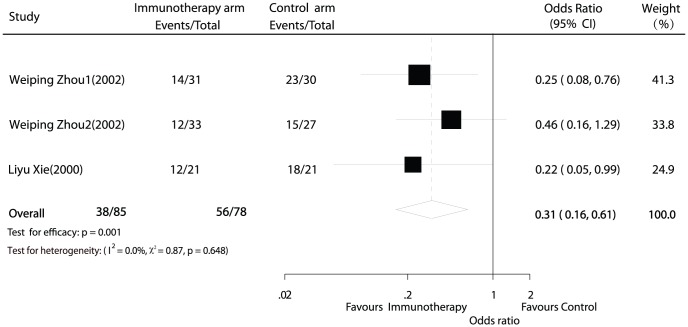
Comparison of 3-year recurrence between hepatocellular carcinoma therapy and non-immunotherapy postoperation therapy.

Information on 3-year recurrence was available for 2 trials (163 patients) [15], [16]. Among the 94 recurrence cases, HCC recurred in 38 (44.7%) adoptive immunotherapy patients compared with 56 (71.8%) controls within 3 year. Again, the meta-analysis demonstrated a significant reduction in the risk of 3-year recurrence in the group undergoing adoptive immunotherapy with a pooled OR estimated at 0.31 (95% CI 0.16 to 0.61; p = 0.001; figure 3). There was no evidence of heterogeneity among individual studies (p = 0.648; I^2^ = 0%).

### Survival

**Figure 4:**
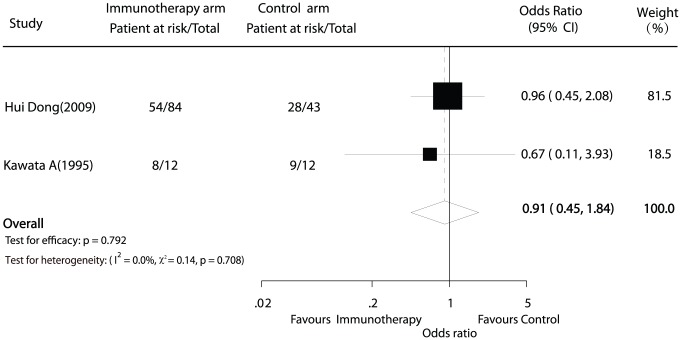
Comparison of 3-year overall survival between hepatocellular carcinoma therapy and non-immunotherapy postoperation therapy.

Information on 3-year overall survival was available for 2 trials [8], [18] including 151 patients. No statistically significant difference was observed between adoptive immunotherapy and no adjuvant treatment for 3-year survival (OR = 0.91; 95% CI, 0.45 to 1.84; *P* = 0.792; Figure 4). There was no evidence of heterogeneity among individual studies (p = 0.708; I^2^ = 0%).

## Discussion

Our analysis confirms that adoptive immunotherapy is a safe, feasible treatment that can decrease recurrence and improve recurrence-free outcomes for postoperative HCC. However, overall survival is not affected.

One reason is that tumor could escape the host immune surveillance and is difficult to cure. The human immune responses against tumor are mainly dependent on the cellular immunity. The host immune response in HCC patients is significantly suppressed. The cytotoxic cell number and cytotoxicity are necessary for efficient immunotherapy of tumors. CIK and LAK cell immunotherapy are two of the most efficient approaches of HCC. Some studies indicated that CIK or LAK cell transfusion was safe and efficient to treat HCC [19]–[21].

Early animal experiments showed that immunotherapy was effective in eliminating micrometastases [22]. Patients with fewer burdens of micrometastases benefited more from immunotherapy than those with larger burden of micrometastases [7]. Most of the local recurrence of postoperative HCC occurs during the 6 months after the operation, adoptive immunotherapy can eliminate these residual cancer cells and destroy the proliferating cancer cells. So immunotherapy could decrease recurrence rate and improve recurrence-free outcome for postoperative HCC [23].

Immunotherapy can only eliminate micrometastases and can not prevent multi-center recurrence of HCC [7]. Therefore, early survival may differ but overall survival may not differ significantly. Since recurrence is just one of the causes of death in HCC (the other causes of death include liver failure, gastrointestinal bleeding, hepatic encephalopathy, renal failure, infection) [24], different effective treatments for recurrence is another reason of no difference of the overall survival.

Postoperative adjuvant TACE appears to be promising for HCC paticents with multiple nodules >5 cm or vascular invasion, so immunotherapy may be used in combination with TACE [25]. Interferon (IFN) has a significant beneficial effect after curative treatment of HCC in terms of both survival and tumor recurrence, so immunotherapy may be combined with IFN [26]. More studies are needed to evaluate the efficacy of such treatment strategies.

A study by Shi et al. (2004) indicated that CIK cell treatment can decrease HBV viral load [19]. A high viral load is an independent risk factor for recurrence after resection of HBV-related HCC [27]. So CIK treatment of HCC patients with HBV infection could be a promising treatment strategy. This is of particular interest since CIK treatment is typically not considered in patients with active hepatitis A, B, or C infection.

Persistent fever was the only severe side effect observed in patients receiving immunotherapy. As a matter of fact, immunotherapy may ameliorate some of the symptoms: patients had increased appetite, improved sleep, gained body weight, and pain relief [19]. Hence, immunotherapy may also improve the quality of life of postoperative patients.

There are some limitations in our study. First, all six trials included in the analysis were conducted in Asia, with two studies published only in the Chinese language. The total sample size is not very large. The follow-up time was not sufficiently long. Some of the studies did not even report the follow-up time, Child-Pugh score, tumor size, background liver diseases and alpha-fetoprotein (AFP). Patients with information on 3-year overall survival was limited (151 patients), and that might cause no difference was observed between adoptive immunotherapy and no adjuvant treatment for 3-year survival.

The reliability of this systemic review might also be influenced by other factors. For example, not all the included studies reported clinic random allocation concealment, so this meta-analysis may have distribution and implementation bias.

Clinical studies with CIK cells are still in their infancy and only involve a relatively small number of patients in most of these studies. The relatively robust and simple cell culture procedures to expand CIK cells have enabled this approach of adoptive cellular immunotherapy to be widely studied. Based on the encouraging experimental and clinical evidence currently available, randomized clinical trials are justifiable and should be done under stringent compliance with the CONSORT principles. This will certainly involve a large number of patients in order to demonstrate statistical significance for a modest degree of outcome superiority. Such studies are urgently needed in order to provide unequivocal evidence of the clinical usefulness of immunotherapy, so as to enable its integration into cancer treatment protocols to improve overall survival [28].
